# Patients’ and Clinicians’ Visions of a Future Internet-of-Things System to Support Asthma Self-Management: Mixed Methods Study

**DOI:** 10.2196/22432

**Published:** 2021-04-13

**Authors:** Chi Yan Hui, Brian McKinstry, Olivia Fulton, Mark Buchner, Hilary Pinnock

**Affiliations:** 1 Asthma UK Centre for Applied Research Usher Institute University of Edinburgh Edinburgh United Kingdom; 2 Tactuum Ltd Glasgow United Kingdom

**Keywords:** asthma, supported self-management, telehealth, mobile application, internet-of-things

## Abstract

**Background:**

Supported self-management for asthma reduces acute attacks and improves control. The internet of things could connect patients to health care providers, community services, and their living environments to provide overarching support for self-management.

**Objective:**

We aimed to identify patients’ and clinicians’ preferences for a future internet-of-things system and explore their visions of its potential to support holistic self-management.

**Methods:**

In an exploratory sequential mixed methods study, we recruited patients from volunteer databases and charities’ social media. We purposively sampled participants to interview them about their vision of the design and utility of the internet of things as a future strategy for supporting self-management. Respondents who were not invited to participate in the interviews were invited to complete a web-based questionnaire to prioritize the features suggested by the interviewees. Clinicians were recruited from professional networks. Interviews were transcribed and analyzed thematically using PRISMS self-management taxonomy.

**Results:**

We interviewed 12 patients and 12 clinicians in the United Kingdom, and 140 patients completed the web-based questionnaires. Patients expressed mostly wanting a system to log their asthma control status automatically; provide real-time advice to help them learn about their asthma, identify and avoid triggers, and adjust their treatment. Peak flow (33/140, 23.6%), environmental (pollen, humidity, air temperature) (33/140, 23.6%), and asthma symptoms (25/140, 17.9%) were the specific data types that patient most wanted. Information about asthma and text or email access to clinical advice provided a feeling of safety for patients. Clinicians wanted automated objective data about the patients’ condition that they could access during consultations. The potential reduction in face-to-face consultations was appreciated by clinicians which they perceived could potentially save patients’ travel time and health service resources. Lifestyle logs of fitness regimes or weight control were valued by some patients but were of less interest to clinicians.

**Conclusions:**

An automated internet-of-things system that requires minimal input from the user and provides timely advice in line with an asthma action plan agreed by the patient with their clinician was preferred by most respondents. Links to asthma information and the ability to connect with clinicians by text or email were perceived by patients as features that would provide a sense of safety. Further studies are needed to evaluate the usability and effectiveness of internet-of-things systems in routine clinical practice.

## Introduction

Asthma is a chronic disease, affecting 235 million people worldwide [1]. Supported self-management reduces emergency use of health care resources, improves asthma outcomes, and reduces morbidity [2-4]. The PRISMS (Practical Systematic Review of Self-Management Support for Long-term Conditions [5]) taxonomy consists of a 14-item list of strategies that have been used to support self-management in long-term conditions and which readers can select according to applicability to their context. In the context of asthma, these strategies include information about the condition, an action plan agreed upon between the patient and their clinician, self-monitoring of asthma status with feedback, recording of physiological measures, use of equipment, lifestyle, and social support. Technology can help support self-management [6], and many patients are interested in a broad range of self-management support strategies, with a seamless link to their clinician if needed [7]. The internet of things (IoT)[8] and wireless networks such as The Things Network (TTN; [9]), long-range wide area networks (LoRaWAN), Wi-Fi, and mobile networks, enable asthma status to be logged automatically by smart devices (eg, smart inhalers, smart peak flow meters, smart watches). Long-life invisible environmental sensors with embedded intelligence supported by Raspberry Pi [10] and Arduino [11] can measure indoor and outdoor environmental triggers which can be correlated to asthma logs that alert patients to changes status, give real-time advice on self-management, and can share data with health care advisors if necessary.

The IoT is a giant system comprising networks linking web-based services and clouds with online sensors and actuators. Sensors are able to communicate with each other to make distributed intelligent decisions in real time [12-15] An app or webpage can be provided to give users a *door* to interact with the system (eg, to view data dashboards; to receive advice; to receive reminders to collect data, reorder medication, or make appointments with their clinician). The IoT health care network has defined an IoT topology [16], which is an architecture and platform that can be used for diagnosis, personalized medication, emergency service, home rehabilitation, remote surgery remote monitoring, and self-management of conditions such as diabetes [17-20], hypertension [18,21], and asthma [18,22,23]. The implementation of 5G IoT will increase data transmission speed and reduce the transmission latency, allowing a faster and seamless service [16]. IoT interconnectivity between patients and their health care providers, the community services, and their living environment can be used to provide overarching and personalized patient self-management support.

There are many asthma-related smart devices in the market (eg, smart inhalers [24], smart spirometers and peak flow meters [25], respiratory rate sensors, wearable sensors that detect wheezing and sleeping patterns [26-28], and digital fraction of exhaled nitric oxide meters [29]), some of which have laboratory-proven accuracy [24,29,30]. Few, however, are able to be personalized to a patient’s clinical profile, social preferences, and environmental context or integrated with a patient’s electronic health records. Mobile systems that connect asthma smart devices and pull data from electronic health records [22,23] and apps that support asthma self-management [31] have been developed, but often, technology researchers focus on novelty and ensuring acceptability of their technology [32], rather than exploring the breadth of functionality that could support patients’ everyday life of living with a condition and meet the demands of clinicians providing routine clinical care [33]. In contrast, clinical research typically focuses on evaluating older, established digital health technologies and their impact on patient health outcomes [34,35]. To our knowledge, there is no research that explores which IoT features are desired by patients and clinicians in the context of asthma self-management. We, therefore, aimed to explore the perspective of patients and clinicians on which self-management features (as defined by the PRISMS taxonomy) they would want in a future IoT system.

## Methods

### Ethical Approval

This mixed methods study was conducted between May 2019 and January 2020, with the approval of the National Health Service (NHS) London Fulham Research Ethics committee (ref 19/LO/0703). The study was sponsored by the University of Edinburgh and the NHS Lothian (Academic and Clinical Central Office for Research and Development). All participants were fully informed about the study and provided their consent.

### Design

We used qualitative interviews and a web-based questionnaire to explore patients’ and professionals’ vision for future IoT features. We adopted an exploratory sequential design [36], using qualitative interviews with purposively selected patients and clinicians to identify the preferred IoT features, which in turn were used to inform a web-based questionnaire in a wider asthma patient community enabling triangulation of interview findings.

### Patient and Clinician Recruitment

#### Patient Recruitment

We included UK-based adult patients who were actively using treatment for asthma [37] and excluded anyone who needed carers’ support to manage their asthma. We recruited patients from volunteer databases and asthma charities social media. We identified patients in the Scottish Health Research Register (SHARE) [38], Register for Asthma Research (REACH), and Asthma UK volunteer database [39]. People registered in these databases have given consent to be contacted about asthma research. Eligible patients were invited with emails sent by SHARE, REACH, and Asthma UK, which included a recruitment link on behalf of the research team. We also posted advertisements on Asthma UK and Asthma UK Centre for Applied Research (AUKCAR) Facebook and Twitter that included a recruitment link. People who were interested in the study used the link to register their interest. They were asked to read the information leaflet, confirm their eligibility, provide basic demographics, and give consent to be contacted (via the contact details they provided) in order complete the registration.

#### Sampling for Qualitative Interviews

We purposively recruited a maximum variation sample of 12 patients to take part in individual interviews. Sampling was based on age (16-25 years, 26-45 years, 16-65 years, 65 years or older); ownership of an action plan (or not); duration of asthma (diagnosed <6 months, 6-12 months, 1-10 years, >10 years); hospital admission in the previous 12 months (or not); confidence with using technology (ability to download apps by themselves, need help, never tried).

#### Clinician Recruitment

We recruited health care professionals from primary and secondary care through newsletters and social media (the NHS Research Scotland Primary Care Network and professional bodies such as the Primary Care Respiratory Society and the NHS Lothian Respiratory Managed Clinical Network). We also approached individual professionals who were actively involved in clinical care or research through personal networks.

### Data Collection

#### Think-Aloud Qualitative Interviews

We adopted a think-aloud approach [40] to explore patients’ and professionals’ preferences on the future design and utility of IoT systems to support asthma self-management.

**Figure 1:**
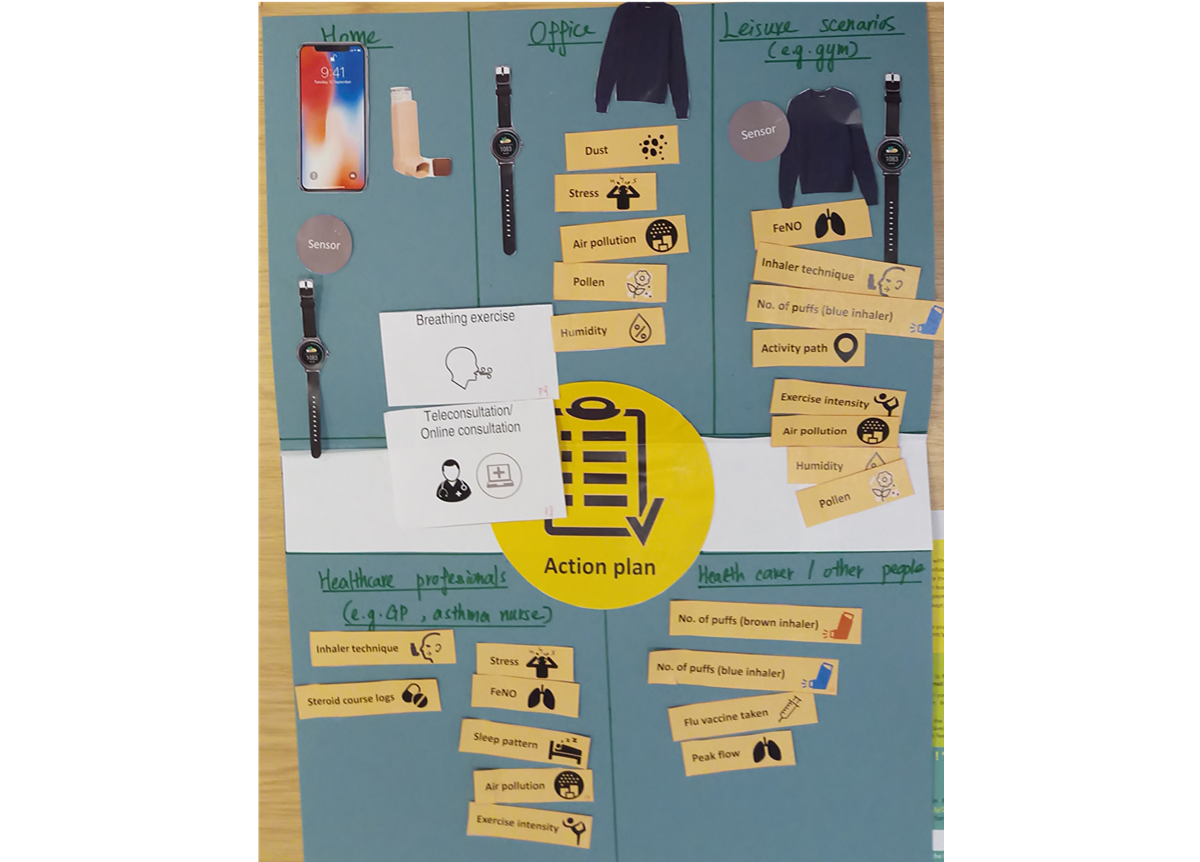
An example of a task completed by a participant.

We asked patients to complete 2 tasks in the interview. For task 1, we provided a list of the most wanted app features (Multimedia Appendix 1) identified in our previous study [7] and asked patients to think about their previous usage of asthma apps and their previous asthma self-management experience to decide the features that they would want to be included in a future IoT system, and how often they think they would use them. For task 2, we provided images of current and future potential smart devices in the market (smart inhaler, smart peak flow meter, smart fabric, smart watch, voice assistant, smart purifier, human robot, and robot pet) and available data and asked patients to discuss their future potential. Specifically we asked them to use the images to create a IoT system that they would want to support their daily self-management, and if, how, and when they would use each device for self-monitoring and what data (if any) they would want to be able to send to their health care professionals and their health carers, for example, parents or spouse. (Figure 1 is an example of a task completed by a participant.)

At the end of each task, participants were asked to add any features, data, or smart devices that they thought would be useful but that were not included in the list or images (or were not yet available).

Professionals were asked to think about their previous experiences providing care for people with asthma and select the features from the list [7] that could support self-management and data that they would like to receive in order to assist their consultations with patients.

Finally, we showed participants a prototype app (an interface app which included many wanted features [7]) to stimulate their thoughts on how they would like to interact with a self-management support IoT system (Multimedia Appendix 2).

#### Development of the Web-Based Questionnaire

The web-based questionnaire aimed to quantify the features wanted by interviewees and their perceived usefulness enabling us to triangulate the findings. Participants’ feedback in the qualitative interview informed the features listed.

The questionnaire (Multimedia Appendix 3) collected basic demographic information (age group and gender) and asked questions regarding what data that they would want an IoT system to collect and where they would want the data to be collected. The flow of questions reflected the sequence that we used in the interviews. To reduce instances of missing data, each question had to be completed before progressing to the next. First, we offered a list of features based on our previous research [7] and the interview responses. Survey participants were asked to select the top 5 types of data that they would like collected by the IoT system and to prioritize them. A free-text option was provided. Second, we asked participants to choose where it would be most useful for the technology to collect these data. The choices were informed by the interviews—“home,” “at work/office/school,” “the place where they are at leisure activities that they do regularly (eg, gym, running, etc),” and “others.” If participants chose the leisure option, we asked the participants to specify which leisure scenario they thought was relevant to that data type. We offered a variety of activities informed by the interviews and a free text option.

#### Administration of the Web-Based Questionnaire

We used Bristol Online Survey, a secure web-based survey platform that complies with ISO27001 information security standards and the General Data Protection Regulation, to build the web-based questionnaire and collect data. To test content validity and readability, we invited a patient volunteer and an independent researcher to try out the questionnaire independently. Their feedback was incorporated in the questionnaire before it was sent to participants. Potential participants who had not been selected for an interview and who had given permission to be contacted regarding the survey were emailed a participant information sheet and a link to the questionnaire.

### Data Analysis and Synthesis

#### Qualitative Analysis

Interviews were digitally recorded, transcribed, and coded in NVivo (version 12; QSR International). We used PRISMS taxonomy [3] as a framework to categorize the IoT features (themes) that emerged from the interviews. In addition, our thematic analysis explored issues of particular importance to the participants. One reviewer (CYH) coded 2 interviews (patient, professional) independently, and another reviewer (HP) reviewed the coded transcriptions to standardize the coding, which was then applied (by CYH) to all the transcriptions. CYH (reviewed by HP) coded features suggested by patients and professionals separately and extracted the features, which were then combined in tables for comparison. We categorized the individual assessments of the features as would “always use,” “often use,” “use when needed,” or “less likely to use” according to the words used in the interviews (Table 1). In our previous study [7] on self-management features wanted by patients’ and clinicians’, no new app features were generated after 15 interviews. We estimated that a sample size of 12 would be likely to achieve data saturation and was within our resources for conducting individual interviews.

**Table 1:** Defining patients’ perceptions of potential future usage.

Usage	Examples of words used by patient interviewees
Always use	“always use,” “would always use,” “very helpful,” “would be good,” “more important,” “definitely a must”
Often use	“would often use,” “would be good,” “really/extremely useful,” “it might be useful,” “something that I’d probably use often, if I could, or if I can”; “once a month,” “quite/might be/ useful,” “might interest me”
Use it when needed	“safety net,” “absolutely need it,” but like “rare occurrences,” “once a year,” “very important just to remind yours occasionally,” “it wouldn’t be like every day,” “a bit of a waste of time if you have that popping up every day,” “check in on regularly”
Less likely to use	Unlikely to use because “I know what to do” “the factors do not affect me”, “the feature is already provided by the current practice”, “no need to have this feature as an extra”; or they “hope not to use this feature very often” or “would not rely on it”; or they “haven’t used it before”.
Not applicable	“Does not apply to them”; “no point in it”

#### Quantitative Analysis and Triangulation of Findings

We used descriptive statistics to analyze patients’ preference ratings for the data they would like a future IoT system to collect and in which circumstances the system would be most useful. We used data from the survey to quantify the features wanted by interviewees and their perceived usefulness.

#### Interpretation

The findings, data synthesis, and interpretation were discussed regularly within the multidisciplinary study team, which included a patient representative, technology developer, health care professionals, and researcher. The researcher had an engineering and technology background and clinical research experience with patients and clinicians in asthma app development.

## Results

### Participants

#### Patients

Invitations were sent by email to 572 patients (SHARE n=211; Asthma UK n=220; REACH n=141). Advertisements were posted to the Asthma UK’s Facebook and Twitter (185,800 followers) and AUKCAR Twitter (1224 followers). Of 362 patients who expressed interest in the study, 297 (82.0%) were from Asthma UK’s social media, email, or website; 42 (11.6%) were from the AUKCAR Twitter; 10 (2.8%) were from SHARE; 3 (0.8%) were from REACH, 2 (0.6%) were recommended by their hospital or a relative, 8 (2.2%) received an e-mail invitation from Asthma UK and others responded to our advertising. Of the 362 patients, 12 were selected for an interview, and the remaining 350 were invited to complete the web-based questionnaire: 3 rejected the invitation (2 were no longer available to take part and 1 declined when they realized there was no payment), and 29 were undeliverable emails. Thus, 318 were sent the web-based questionnaire link, of whom 140 (44.0%) completed the questionnaire. Of the 140 participants, 139 participants (99.3%) completed the questionnaire within 15 minutes, and 1 person (0.7%) appeared to take 3 hours and 42 minutes to complete the questionnaire (most likely because the *thank you* page was not closed after completion). There were no instances of missing data.

Of 152 participants (12 interviewees and 140 web-based questionnaire respondents), most (74/152, 49%) were 46 years to 65 years of age, 69.1% (105/152) were female, 54.6% (83/152) had an action plan, 75.0% (114/152) had been diagnosed with asthma for more than 10 years, 12.5% (19/152) had been admitted to hospital in the previous 12 months, and 86.2% (131/152) were confident that they could download apps themselves (Table 2).

**Table 2:** Characteristics of patient participants.

Patient characteristics			Interviewees (n=12), n (%)		Questionnaire respondents (n=140), n (%)
**Age (years)**					
	16-25	3 (25.0)		2 (1.4)	
	26-45	2 (16.7)		39 (27.9)	
	46-65	3 (25.0)		71 (50.7)	
	>65	4 (33.3)		28 (20.0)	
**Gender**					
	Female	8 (66.7)		97 (69.3)	
	Male	4 (33.3)		42 (30.0)	
	Prefer not to say	0 (0.0)		1 (0.7)	
**Action plan ownership**					
	Yes	4 (33.3)		79 (56.4)	
	No	3 (25.0)		16 (11.4)	
	No asthma action plan but I have been told what to do	5 (41.7)		45 (32.1)	
**Diagnosed with asthma**					
	Less than 6 months	0 (0.0)		1 (0.7)	
	Between 6 months and 1 year	0 (0.0)		1 (0.7)	
	Between 1 year and 10 years	4 (33.3)		32 (22.9)	
	More than 10 years	8 (66.7)		106 (75.7)	
**Admission to the hospital because of asthma in the last 12 months**					
	No	8 (66.7)		125 (89.3)	
	Yes and now my asthma care is provided by general practitioner/asthma nurse	1 (8.3)		5 (3.6)	
	Yes and I am still attending the hospital (specialist) clinic	3 (25.0)		10 (7.1)	
**App download experience**					
	I download apps by myself	12 (100)		119 (85.0)	
	I usually ask someone to download apps for me	0 (0.0)		5 (3.6)	
	I have never downloaded an app	0 (0.0)		16 (11.4)	

#### Professionals

Twelve professionals were recruited from primary, secondary, and tertiary care in the United Kingdom. All provided care for people with asthma, and some had research experience using digital technology to monitor patients’ medication use and symptoms for respiratory patients (Table 3).

**Table 3:** Characteristics of professional participants.

Professional role	n	Experience^a^	Additional description
General practitioner	2	>8 years	Primary care: respiratory lead n=1; accident & emergency experience n=1
Asthma nurse	2	>20 years	General practice asthma-trained nurse n=2
Pharmacist	4	1-20 years	Respiratory pharmacists n=3; community pharmacist n=1 (reviewing patients during asthma admissions or when referred by general practitioner or asthma nurse; checking inhaler technique, choosing devices, addressing medication adherence)
Consultant chest physician	1	—^b^	Secondary and tertiary care (severe asthma center and community lead)
Asthma pediatrician	3	—^b^	Lead consultant in a pediatric asthma service n=1; Using smart inhaler n=1; 30 years of pediatric asthma research experience n=1 (looking after children with a range of asthma severities, conducting face-to-face consultation, determining patients’ symptoms, making management plans, offering advice to general practitioners, and reviewing test results)

^a^Experience seeing patients with asthma on a regular basis.

^b^Information not available.

### Mixed Methods Assessment: Features and Their Perceived Usefulness

#### Interview Themes

Perceptions of the 12 patients about the potential usefulness of features were mapped to the PRISMS taxonomy (Table 4; the full version can be found in Multimedia Appendix 4). Features wanted by patients reached saturation within 10 interviews, and we stopped sampling at 12 interviews.

Patients decided the usefulness of potential IoT features based on their own past asthma self-management experiences (eg, ownership or use of action plan and smart peak flow meter, their relationship with their clinicians, medication usage, use of emergency services), their asthma triggers, their curiosity about what affected their asthma, the severity and control of their asthma condition, and what they considered (or had been told by professionals) was best practice for asthma.

Monitoring, supported by feedback advice, was the feature that most patients wanted to see in an IoT system. Information about asthma and an action plan were also priorities. Flexible access to follow-up advice with a general practitioner or asthma nurse by text or email service were “safety net” features that most patients thought would create a sense of “staying connected” with clinicians, although they stated they would only use it when needed. One patient with hearing problems who struggled to communicate during an exacerbation wanted a panic button to automatically text for emergency help. Perceptions on monitoring of control, and feedback are presented in more detail below. Other features were wanted by patients but were lower priority (Multimedia Appendix 4).

**Table 4:** Summary of the potential usefulness of features mapped to the PRISMS taxonomy [5].

Theoretically based support	Features	Patient	Clinicians
Information about asthma and available resources	Information about asthma management	Online information was of interest, ideally personalized to clinical context and individual situation (eg, broad range of information for newly diagnosed, safety net information for experienced patients)	Professionals considered that reputable information such as inhaler technique videos, treatment information, why and how the medication should be taken were important for patient self-management
Provision of action plan	What to do when condition gets worse	Most patients wanted a (digital) action plan to remind them what to do when they forgot the agreed actions when their conditions were getting worse	Most clinicians suggested an action plan to remind about medication adjustment and agreed actions if patients’ condition was getting worse
Regular clinical review	Routine review reminder; remote options.	Most patients wanted reminders for the yearly review. Some preferred web-based or teleconsultation consultation for regular reviews to save travel time	Most professionals thought reminders would encourage attendance, and agreed remote consultations were convenient though not always clinically appropriate
Monitoring condition with feedback	Logging asthma symptoms, peak flow, and medication use.	Most patients wanted automatic logging with intelligent feedback and the facility to transfer data to the hospital or general practitioner practice. Reminders could be useful though ideally only generated when their asthma was bad. Some patients wanted alerts when they had increased use of rescue inhaler	Most clinicians thought objective data would help them assess status and help patients understand their triggers. They were skeptical that reminders would improve adherence to logging. Flagging excess or increasing use of recuse medication could alert patients and professionals to poor control
Practical support with adherence	Medication reminders and support	Some patients thought reminders to take medication were useful if they were busy or forgetful but would not change opinions. Automatic prompting reordering of medication was wanted. Most patients wanted flu vaccine alerts	Clinicians wanted medication adherence logs and agreed with low-medication alerts to facilitate reordering. Warning about overordering were also important. Flu vaccination reminders should pop up in both patients and clinicians’ system
Provision of equipment	Smart devices	Most patients wanted to try smart devices (inhalers, peak flows, activity trackers)	Clinicians generally interested in how the whole technology system, as opposed to how individual smart device can support patients
Provision of easy access to support when needed	Panic button for emergency	A few patients suggested this would be helpful because it could be difficult to speak during exacerbations	Clinicians generally considered this was a duplicate emergency system, though might be useful for brittle high-risk asthma
Communication with health care professionals	Emails, texts, and WhatsApp messages	Most patients wanted flexibility to ask quick follow up questions, and a patient with hearing problems found WhatsApp useful	Clinicians agreed with a flexible approach to reviews, including text services for quick follow up questions, though resources would be needed
Training for everyday activities	Air pollution/pollen high alert	Most patients wanted environmental features, and some young patients (16-25 years old) suggested it could identify triggers and help them to plan their day	Clinicians were less interested in these data, though some thought environmental information could help patients to understand (and avoid) triggers
Training for self- management activities	Incorrect inhaler technique alert	Most patients thought it was good to be prompted when their inhaler technique was incorrect	Most clinicians wanted inhaler technique checks with real-time alerts for patients recorded for discussion at a review
Training for psychological strategies	Breathing exercise	Some patients wanted breathing exercises to keep themselves calm and considered it would help their asthma	Most clinicians were keen to encourage patients to do breathing exercises to improve asthma symptoms
Social support	Social media and alerts	One patient wanted a friend alerted when she was admitted to the hospital	One clinician suggested a social media page to enable sharing of experiences
Lifestyle advice and support	Physical activity and weight loss	Some patients wanted to connect asthma logs with their activity tracker, while others would use a weight watching facility	One clinician suggested an individualized fitness program for people with severe asthma, and 2 clinicians wanted individualized weight management plans

#### Survey Findings and Triangulation With Interview Themes

**Figure 2:**
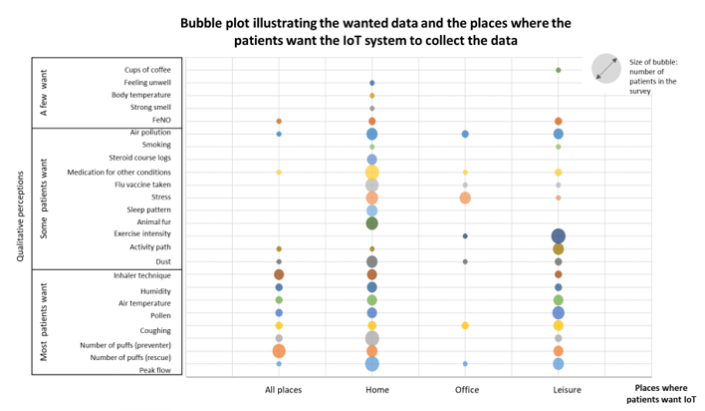
Information that patients want internet-of-things system features to capture. FeNO: fractional exhaled nitric oxide.

The responses to the survey are illustrated with a bubble plot (Figure 2). In keeping with the priorities of the interviewees, the largest bubbles represent monitoring of symptoms (prioritized by 25/140, 17.9%) and peak flow measurement (33/140, 23.6%). For most respondents (24/32, 75.0%), this was a task to be undertaken at home, though some considered that symptom monitoring could be useful at home, work, or leisure locations. Monitoring environmental asthma triggers (pollen, humidity, air temperature) was prioritized by 23.6% (33/140).

### Qualitative Perceptions of Specific Features

#### Smart Devices for Monitoring Asthma Control

Some patients explained that a peak flow was “useful” and “needed” to help clinicians assess their asthma. Most professional interviewees agreed with this and thought that a log of peak flows, symptom scores, and medication use would engage patients and could inform assessment of control and management strategies.

I see particularly important is logging asthma symptoms and medication use. Peak flow, maybe less so, but the symptoms and medication use I think are very important. although peak flow is far from perfect, it just shows that the patient has engaged to some degree, that they’re seeing differences, whether it’s in the morning or evening or when they...after exercise and they’re engaged with that, so I know when I ask them some questions about their symptom experience that they’re thoughtful about it.

Health care professional 7, general practitioner

However, in contrast to the priority attached to logging, most patients acknowledged that in reality they checked their peak flow “rarely” or “only when their asthma was getting worse.” The reasons for not measuring every day were varied. Some “forgot,” while others felt “weird doing it in front of people,” but many suggested it was unnecessary as they knew their asthma and could assess status by how they felt.

Not very often. I do sometimes, if I get chest infections or if I get tight chested or if it’s not, if I just feel there’s something wrong, then I test what my peak flow is, just to see what it is.

Patient 4, 26-45 years old, male

I’m a big believer in logging things so that if you’ve got a history... I mean to log things, but I forget, so it would be good to have something there.

Patient 5, >65 years old, male

Several suggestions were made about how an IoT system could help overcome this discrepancy. A word that was used frequently was “automatic.” Most patients wanted an IoT system that could automatically log their asthma condition. They were interested in trying smart devices (eg, smart peak flow meter); some explicitly mentioned that this would enable them to capture data automatically though they were not clear how the devices could capture their asthma status without some effort on their part. A device with an automated data transfer feature still requires the user to blow the air into the meter, and patients acknowledged that to do this regularly would require motivation such as a request from researchers or their clinician. Patients had different opinions on using voice assistants, smart watches, smart fabrics, smart purifiers, human robots, and robot pets to collect additional data automatically:

Smart peak flow would be the way forward I think, it really would, because it’s recording it (my peak flow). If you say to me record it every day, I would record it every day...I very rarely use it (the current mechanical peak flow). But if this smart peak flow is going to record my peak flow at that particular time each day or twice a day, that’s only going to be good to help me and to help my doctor understand, you know.

Patient 2, >65 year old, male

It would be good to have something that I could just quickly tell it what’s happening. Probably not when I’m having an asthma attack because I can’t talk.

Patient 1, 46-65 years old, female

I don’t have my inhalers with me and my other sensors in the office, so for me, given I’m going in and out of meetings and various other things and travelling about and stuff, it’s probably easier if I just have the watch and any clothing or something with sensors on it that can do it, in an ideal world.

Patient 4, 26-45 years old, male

(Voice assistant) It’s not very secure, I don’t like that.

Patient 5, >65 years old, male

How the data were used was an important motivator. Most patients said it was “good” or even “essential” that data from smart peak flow meters and inhalers were transferred automatically to the system so these logs could be correlated with other data and displayed graphically on a mobile phone. Some suggested that they would log peak flows if their clinicians asked them to do so.

Clinicians were keen to see a record of medication use to enable assessment of adherence. They favored automated logging of this information via a smart inhaler as that was perceived to be more accurate.

It would be very useful if the patient is logging their asthma symptoms and peak flow, medication use...because that’s then helping us to adjust on treatment. I would love to know if they were taking it (prescribed medication) every day like they’re telling me they are.

Health care professional 4, prescribing support pharmacist

#### Feedback and Advice

Patients in the interviews suggested a broad range of information and advice that could be usefully provided through an IoT system such as a timely alert when their asthma control changed; the amount of medication to be taken according to asthma control to reduce medication side effects; the numbers of doses taken and remaining in the inhaler, ideally with an option to order a repeat prescription when the medication was running low; and correct inhaler technique.

The clinician interviewees concurred with these priorities, especially the need for detecting poor inhaler technique and linking to information about correct use of an inhaler. They also saw value in feedback supporting treatment adjustment according to an action plan.

#### Environmental Data

Outdoor environmental data such as pollen, humidity, and air temperature were considered “good to know” and “useful” data by many patients. Patients who were newly diagnosed with asthma wanted to learn which environmental factors affected their asthma, whereas those who already knew what triggered their asthma wanted to use daily environmental data to plan their day.

(The outdoor environmental data) could maybe suggest like how likely they are to be actual triggers as opposed to me just thinking. Then if it suggests that I’m really triggered by something then I could put more effort to avoid that.

Patient 10, 16-25 years old, female

These data were of less interest to clinicians though some suggested that, together with indoor triggers, outdoor pollution, exercise intensity, weight, peak flow, and symptoms, data could provide real-time feedback and help patients understand which factors affected their asthma in order to avoid them.

## Discussion

### Principal Results

Both patients and clinicians expressed their interest in automated monitoring with real-time feedback within an IoT system that could support a wide range of self-management tasks. In the qualitative interviews, patients mostly wanted the system to log peak flow, asthma symptoms, and environmental triggers (pollen, humidity, air temperature); provide advice on relevant actions or medication adjustment to suit different levels of asthma control; and provide alerts about the number of doses of medication remaining and inhaler technique. The questionnaire responses quantified these preferences with the most wanted features being monitoring peak flow (33/140, 23.6%), environmental asthma triggers (pollen, humidity, air temperature) (33/140, 23.6%), and symptoms (25/140, 17.9%). Clinicians wanted automated objective logs about patient condition that they could access during a consultation. Patients considered that easy access to information and clinical advice, such as text or email communication via the system, provided a feeling of safety, while clinicians appreciated the potential reduction in face-to-face consultations because it would reduce patient travel time and the use of health service resources. Lifestyle logs (fitness regimes or weight control) were wanted by some patents but were of less interest to clinicians.

### Strengths and Limitations

Our study identified the preferred IoT features for patients and clinicians and the type of data that they wanted the system to collect; however, there are some limitations. First, due to limited resources and time, we excluded children under 16 years old from patient interviews though we included experienced pediatricians to provide insights on the needs of children with asthma and their carers. Second, we did not manage to interview any patients who were newly diagnosed with asthma (within the previous year) who may well have had specific needs for information and support. Similarly, all our participants were familiar with downloading and using apps, suggesting that our recruitment strategy of using social media reached a technologically experienced population. Although this approach will have resulted in some perspectives being overlooked, the sample included in the study were demographically diverse, with a range of experiences of living with asthma, and could provide a range of perspectives on the potential of technological support. Increasingly, the global population is becoming more familiar with digital communication. However, we reached data saturation [41,42] in the qualitative analysis, and the web-based questionnaire, which attracted a broader range of patients, provided findings that were consistent with the qualitative data. Third, though the features that participants said that they wanted were related to participants’ past experience, we also used images of emerging technologies and stimulated discussion about novel features that could potentially contribute to managing their asthma.

### Reflexivity

The researchers had engineering and research experience in developing asthma apps. To reduce the influence on the interview findings of the researcher’s background, the coding and interpretation of results were discussed with study team members from different backgrounds and with different experiences, including general practitioners, a patient, and a technology developer. This range of expertise enabled the study to present a balanced interpretation.

### Comparison With Published Literature

IoT is an option to support self-management. Asthma action plans, asthma education, and regular consultations with clinicians are core component of effective self-management; which aims to support patients achieve good control by recognizing when their asthma is getting worse and responding promptly and appropriately [43]. The features that patients wanted from the IoT system resonated with these aims (ie, such as facilitating learning about asthma and its triggers, logging asthma status, providing alerts to highlight deterioration and offering feedback advising on treatment adjustment and other actions). Features such as monitoring how much medication is left and inhaler technique can be delivered by smart inhalers that are already on the market.

There is, however, an anomaly. Most patients wanted features that enabled regular monitoring of peak flow and symptoms, but the literature suggests that this is rarely, if ever, achieved in real life [7]. For example, in the context of chronic obstructive pulmonary disease, patients who are more severely affected by their disease (ie, those who might find monitoring most helpful) were less likely to use communication technologies such as mobile phones, text messaging, email, and video chat [44]. Our interviewees acknowledged this discrepancy by explaining that they actually only logged their asthma control when they were concerned about increasing symptoms, but that, most of the time, they did not see the necessity of monitoring because they already knew the status of their asthma. Reminders are unlikely to solve the problem of nonadherence to logging because, when patients did not complete logs, it was typically intentional and not due to forgetfulness [45]. A solution suggested by both patients and professionals was to simplify data collection by automating the process and increase motivation by providing useful graphical feedback or linking with professional advice, though even this requires some input from the patient. Further advances may require the development of systems that that silently monitor use of rescue medication with a smart inhaler that requires no input by the patient and only alerts patients in the event of unusual behavior patterns (eg, increased usage) with advice from their action plan on jhow to regain control.

Monitoring is also influenced by whether the patients’ clinician is interested in the results. Home monitoring with feedback has shown promise as a self-management strategy for people with hypertension [46] and diabetes [47]. The sense of having the ongoing interest and support of a clinician is a factor in maintaining motivation to self-manage [48]. Our interviewees felt “safe” if they had an easy way (text or email) to contact clinicians when they had concerns or needed clarifications. A recent review [49] of patients with asthma and chronic obstructive pulmonary disease revealed similar preferences for accessible support. Similarly, patients with type 2 diabetes wanted technology that allowed 2-way text communication with their clinicians.[50]. The professional interviewees in our study considered that, when text and email are offered as options, they have the potential to reduce face-to-face consultations and save health care resources.

### Conclusion

An IoT system can encompass the range of components needed to support asthma self-management. Patients and clinicians preferred features that monitor asthma status (preferably using automated silent monitoring), provide timely advice in line with an agreed upon asthma action plan, and allow observation of environmental factors in relation to asthma control. Our technologically literate participants appreciated the ability to connect to asthma information, as well as easy access to clinicians by text or email. Sustained use was acknowledged as a challenge. Large-scale evaluation of usability, health outcomes, and resource implications are needed to realize the potential benefits of silent monitoring connected IoT systems.

## Appendix

Multimedia Appendix 1 Features and screenshots.

Multimedia Appendix 2 Detailed topic guide.

Multimedia Appendix 3 Web-based questionnaire.

Multimedia Appendix 4 Perceived usefulness of wanted feature.

## Abbreviations

AUKCAR: Asthma UK for Applied Research
IoT: internet of things
NHS: National Health Service
REACH: Register for Asthma Research
SHARE: Scottish Health Research Register
