# Supplementary material for: Patients’ and Clinicians’ Visions of a Future Internet-of-Things System to Support Asthma Self-Management: Mixed Methods Study
Source: J Med Internet Res. 2021 Apr 13;23(4):e22432. doi: 10.2196/22432 (PMC8080146; doi:10.2196/22432)
Supplement: Multimedia Appendix 1 [file jmir_v23i4e22432_app1.docx]

Multimedia Appendix 1: Wanted features and screen shots of the prototype A4A+ app: (from left) home, asthma log, log reminder, smart inhaler and text message with practices

| **Screenshots of our prototype app** | | | | |
| --- | --- | --- | --- | --- |
| 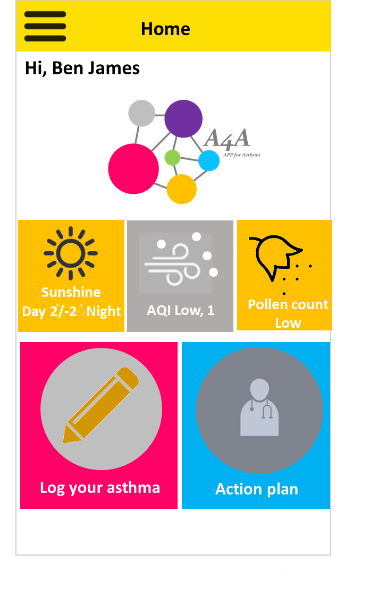 | 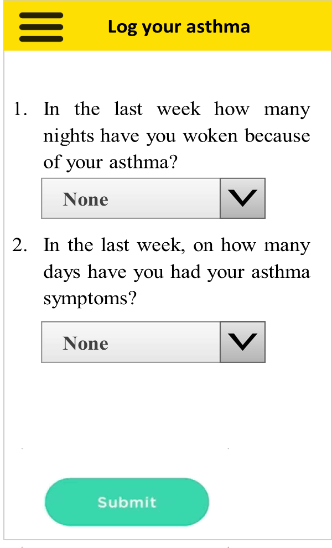 | 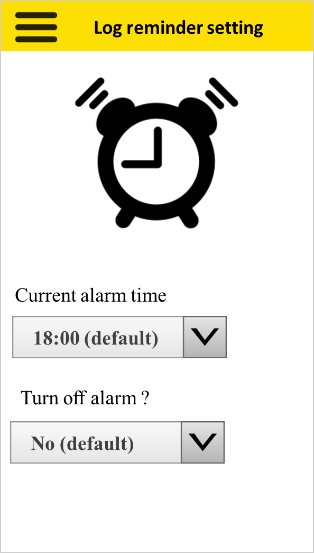 | 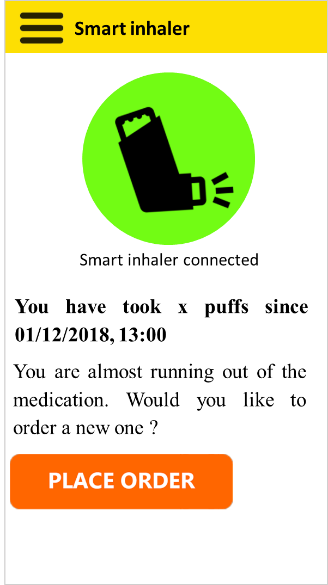 | 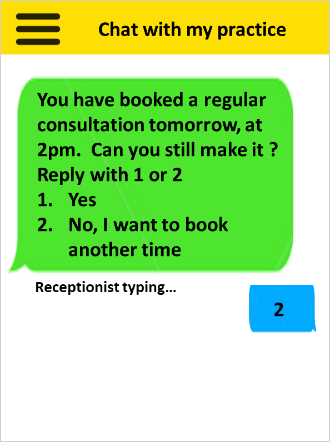 |
| **Wanted features from previous work** [1]  Information about asthma management; teaching patient what to do when their conditions is getting wors; routine review reminder; tele/online-consultation; logging asthma symptoms; peak flow and medication use; log reminder; usual dose alert to patient; medication reminder; medication low reminder (blue/brown) and automatically order repeat prescription; flu vaccine reminder; panic button for emergency (to the duty doctor in the practice, to NHS 111 or 999); follow up conversations with GP/ asthma nurse via emails; text messages and Whatsapp; air pollution/pollen high alert; cross referencing environmental factors and asthma logs to suggest route; cross referencing environmental factors and asthma logs to suggest triggers; incorrect inhaler technique alert; breathing exercise, cross referencing physical activity and asthma logs to suggest fitness trainings; weight and asthma watching. | | | | |
| **Reference**: 1. Hui CY, McKinstry B, Pinnock H et al. Time to change the paradigm? A mixed method study of the preferred and potential features of an asthma self-management app. Health Informatics Journal. 13 Jun 2019:1460458219853381 | | | | |
