# Supplementary material for: Patients’ and Clinicians’ Visions of a Future Internet-of-Things System to Support Asthma Self-Management: Mixed Methods Study
Source: J Med Internet Res. 2021 Apr 13;23(4):e22432. doi: 10.2196/22432 (PMC8080146; doi:10.2196/22432)
Supplement: Multimedia Appendix 2 [file jmir_v23i4e22432_app2.pdf]

Patients and clinicians preferences on internet-of-things (IoT) system to support asthma self-management: a mixed method study from the A4A+ group

Multimedia Appendix 2: Detailed topic guide

We will explore participants' self-management routine, their opinions on the practicalities of using these technologies, trusting the measurements, if/how their use would fit into patient/professional routines and the potential role of emerging technologies in patients' management journey. We will then demonstrate our app to them and ask for their feedbacks on the design and functionality.

Patient participant interview

Open question

- How do you manage your asthma? *(take preventer inhaler in the morning/ attend regular consultation/ take action according to action plan when symptoms is back/ use experience to adjust or use of medication or to decide the actions to be taken/use of peak flow meter)*
- What do you want to see on an app to help you manage your asthma?

We provide the features cards to patients and ask them:

- What features do you think you will use always, often, and less likely to use? Why?
- Any other features that you think is important to help managing your asthma?
- (of the features that they select to use always) how do you want to use these features at home, your office and a scenario that you will always be (e.g. gym)?
- Do you think app or smart inhaler or AI or IoT device is trustworthy tool to diagnosis your asthma and give you advice on what to do when your asthma is getting worse? Why?
- What could be the potential role of technologies such as app, smart inhaler, AI and IoT devices in your asthma self-management journey? *(remind them about their asthma/help them to learn self-managing their asthma/ remind them about regular consultation)*

We demonstrate our app to the participants and ask them:
What do you think about our app? *(features/ user interface/ feel easy to use/ want username and password protection)*

Professional participant interview

Open question

We show the features that patients wanted to them and ask them:

- What do think about the features suggested by patients?
- Any other features that they think can support patients managing their asthma?
- What features do you think are important to patients/ professionals?
  (Of the features that are important for professionals) Which features do you think will improve your assessment to your patients/ which features do you think will save your time?
- If/how the features could be fitted in their practice routine and if/how they could be implemented?
- Do you think app or smart inhaler or AI or IoT device is trustworthy tool to diagnosis patients' asthma and give advice on what to do when your asthma is getting worse? Why?
- What could be the potential role of technologies such as app, smart inhaler, AI and IoT devices in patients' asthma self-management journey?

We demonstrate our app to the participants and ask them:
What do you think about our app? *(features/ user interface/ feel easy to use in their routine practice)*
