# Supplementary material for: Patients’ and Clinicians’ Visions of a Future Internet-of-Things System to Support Asthma Self-Management: Mixed Methods Study
Source: J Med Internet Res. 2021 Apr 13;23(4):e22432. doi: 10.2196/22432 (PMC8080146; doi:10.2196/22432)
Supplement: Multimedia Appendix 3 [file jmir_v23i4e22432_app3.pdf]

A4A+ online questionnaire

Privacy Statement

A4A connected plus study only uses the personal data you have provided to deliver a questionnaire for the purposes of determining people's preference to use emerging technologies to support your asthma management. To do so, we are using Bristol Online Surveys as our questionnaire platform. The information you provide will be used by the University to find out if there are any association between people's preference on the emerging technologies and age, gender, ownership (or not) of an asthma action plan, how long they have had asthma; if/if not under hospital care for their asthma; ability (or not) to download an app by themselves.

We are using information about you because the University has a legitimate interest in carrying out the questionnaire to allow us to analyse how the emerging technology can support your asthma self-management.

We will hold the personal data you provided us for a minimum of 5 years and may be used in future ethically approved research. The information that you provided will be anonymised.

We do not use profiling or automated decision-making processes.

If you have any questions, please contact Dr. Io Hui (research fellow) by phone: 01316503209 or email: io.hui@ed.ac.uk

This Privacy Statement is continued at:

www.edin.ac/privacy

1. I understand that all of the information about me recorded for this project will be anonymised (that is, my name will be removed) and will not affect my confidentiality in any way. If the results of the study are published it will not be possible to identify information about me.
2. I understand that my anonymised data will be stored for a minimum of 5 years and may be used in future ethically approved research.
3. I understand that relevant sections of my data collected during the study may be looked at by individuals from the regulatory authorities and from the Sponsor(s) (NHS Lothian and the University of Edinburgh) or from the/other NHS Board(s) where it is relevant to my taking part in this research. I give permission for those individuals to have access to my records.
4. I give permission for my age, gender, ownership (or not) of an asthma action plan, Asthma experience such as how long they have had asthma; if/if not under hospital care for their asthma; and my ability (or not) to download an app to be used in the analysis of the results.
5. I agree to take part in this study.

I give my consent to the above items ✱ *Required*

○ Yes

About you

To help us to understand how technology can help different people, it will be helpful if we learn a little bit about you

Age ✱ *Required*

Please select no more than 1 answer(s).

- ☐ 16-25
- ☐ 26-45
- ☐ 46-65
- ☐ 65 or over

Gender ✱ *Required*

Please select no more than 2 answer(s).

- ☐ Female
- ☐ Male

Have you been given an asthma action plan? ✱ *Required*

Please select no more than 1 answer(s).

- ☐ Yes
- ☐ No
- ☐ No asthma action plan but I have been told what to do

How long have you been diagnosed with asthma? ✱ *Required*

Please select no more than 1 answer(s).

- ☐ Less than 6 months

- ☐ 6 months – 1 year
- ☐ 1 year – 10 years
- ☐ More than 10 years

Have you been admitted to hospital because of your asthma in the last 12 months ? ✱ *Required*

Please select no more than 1 answer(s).

- ☐ No, I have not been admitted to hospital because of my asthma in the last 12 months
- ☐ Yes, I have been admitted because of my asthma in the last 12 months, but now my asthma care is provided by GP/asthma nurse
- ☐ Yes, I have been admitted because of my asthma in the last 12 months, and I am still attending the hospital (specialist) clinic

Do you download your own apps to your devices? ✱ *Required*

Please select no more than 1 answer(s).

- ☐ Yes
- ☐ No, I usually ask someone to download for me
- ☐ No, I have never downloaded an app

Question 1

In the interview, our patient participants have suggested a list of data that they would like emerging technologies such as smart inhaler, smart peak flow meter and activity tracker to collect data in their daily lives.

Data can be either automatically collected by the app/smart gadget or entered manually or recorded by voice.

They want technologies that help them to know more about their asthma and give advice on their lifestyle activities.

Please select the top five data that you would like the technology to collect to help you know more about your asthma and give advice on your lifestyle activities.

	Data you want the technology to collect ✱ *Required*
1st wanted data	Please select
2nd wanted data	Please select
3th wanted data	Please select
4th wanted data	Please select
5th wanted data	Please select

Question 2

Where / when do you want the technology to collect the data ?

The most wanted feature: [GRID_1_COL1] ✱ *Required*

- ☐ When I am at home
- ☐ When I am in my work/office/school
- ☐ When I am at leisure activities that I do regularly (e.g. gym, running etc.)
- ☐ Other

If you selected Other, please specify:

The 2nd wanted feature: [GRID_2_COL1] ✱ *Required*

- ☐ When I am at home
- ☐ When I am in my work/office/school
- ☐ When I am at leisure activities that I do regularly (e.g. gym, running etc.)
- ☐ Other

If you selected Other, please specify:

The 3th wanted feature: [GRID_3_COL1] ✱ *Required*

- [ ] When I am at home
- [ ] When I am in my work/office/school
- [ ] When I am at leisure activities that I do regularly (e.g. gym, running etc.)
- [ ] Other

If you selected Other, please specify:

The 4th wanted feature: [GRID_4_COL1] ✱ *Required*

- [ ] When I am at home
- [ ] When I am in my work/office/school
- [ ] When I am at leisure activities that I do regularly (e.g. gym, running etc.)
- [ ] Other

If you selected Other, please specify:

The 5th wanted feature: [GRID_5_COL1] ✱ *Required*

- [ ] When I am at home

- [ ] When I am in my work/office/school
- [ ] When I am at leisure activities that I do regularly (e.g. gym, running etc.)
- [ ] Other

If you selected Other, please specify:

Any other data that you would like the technloogy to collect ? ✱ *Required*

- ( ) Yes
- ( ) No

Other data

What other data do you want the technology to collect ?

Other data

Where / when do you want the technology to collect [TEXT] ? ✱ *Required*

- ☐ When I am at home
- ☐ When I am in my work/office/school
- ☐ When I am at leisure activities that I do regularly (e.g. gym, running etc.)
- ☐ Other

If you selected Other, please specify:

Leisure scenarios

If you have said that you want to collect the data in a 'leisure scenarios'. What is the leisure scenarios that you are thinking of, please select? ✱ *Required*

- Gym/ outdoor exercise (running, biking, hiking etc)
- Outdoor activity (e.g. causal walking, jogging, walk a dog etc)
- Sea and water activity (e.g. swimming, diving and kayaking)
- Not Applicable
- Other

If you selected Other, please specify:

Consent to receive a summary of results

I wish to receive a summary of the study findings at the end of the A4A+ study and give consent for the study team to send results and publications to me on the e-mail you are using for this invitation. ✱ *Required*

- Yes
- No

Thank you for taking part in our A4A+ study.

If you have any concerns about the study, please contact Dr. Io Hui: io.hui@ed.ac.uk.

Key for selection options

8.1.a - Data you want the technology to collect

Pollen, humidity, air temperature
Outdoor air pollution
Dust, animal fur or feathers
Indoor air pollution
Peak flow
FeNo
Asthma symptoms(wheeze, tightness in chest, feeling breathless)
Coughing in bed
Coughing in all day
Sleep disturbance
No. of puffs (reliever inhaler)
No. of puffs (preventer inhaler)
Medication used for asthma and other conditions
Medication stock for asthma and other conditions
Inhaler technique
Flu vaccination taken
Oral steroid course log
Exercise intensity and activity path
Stress
Smoking cessation
Weight

8.2.a - Data you want the technology to collect

Pollen, humidity, air temperature
Outdoor air pollution
Dust, animal fur or feathers
Indoor air pollution
Peak flow
FeNo
Asthma symptoms(wheeze, tightness in chest, feeling breathless)
Coughing in bed

Coughing in all day
Sleep disturbance
No. of puffs (reliever inhaler)
No. of puffs (preventer inhaler)
Medication used for asthma and other conditions
Medication stock for asthma and other conditions
Inhaler technique
Flu vaccination taken
Oral steroid course log
Exercise intensity and activity path
Stress
Smoking cessation
Weight

8.3.a - Data you want the technology to collect

Pollen, humidity, air temperature
Outdoor air pollution
Dust, animal fur or feathers
Indoor air pollution
Peak flow
FeNo
Asthma symptoms(wheeze, tightness in chest, feeling breathless)
Coughing in bed
Coughing in all day
Sleep disturbance
No. of puffs (reliever inhaler)
No. of puffs (preventer inhaler)
Medication used for asthma and other conditions
Medication stock for asthma and other conditions
Inhaler technique
Flu vaccination taken
Oral steroid course log
Exercise intensity and activity path
Stress
Smoking cessation
Weight

8.4.a - Data you want the technology to collect

Pollen, humidity, air temperature

Outdoor air pollution
Dust, animal fur or feathers
Indoor air pollution
Peak flow
FeNo
Asthma symptoms(wheeze, tightness in chest, feeling breathless)
Coughing in bed
Coughing in all day
Sleep disturbance
No. of puffs (reliever inhaler)
No. of puffs (preventer inhaler)
Medication used for asthma and other conditions
Medication stock for asthma and other conditions
Inhaler technique
Flu vaccination taken
Oral steroid course log
Exercise intensity and activity path
Stress
Smoking cessation
Weight

8.5.a - Data you want the technology to collect

Pollen, humidity, air temperature
Outdoor air pollution
Dust, animal fur or feathers
Indoor air pollution
Peak flow
FeNo
Asthma symptoms(wheeze, tightness in chest, feeling breathless)
Coughing in bed
Coughing in all day
Sleep disturbance
No. of puffs (reliever inhaler)
No. of puffs (preventer inhaler)
Medication used for asthma and other conditions
Medication stock for asthma and other conditions
Inhaler technique
Flu vaccination taken
Oral steroid course log
Exercise intensity and activity path

Stress
Smoking cessation
Weight
