# Supplementary material for: Patients’ and Clinicians’ Visions of a Future Internet-of-Things System to Support Asthma Self-Management: Mixed Methods Study
Source: J Med Internet Res. 2021 Apr 13;23(4):e22432. doi: 10.2196/22432 (PMC8080146; doi:10.2196/22432)
Supplement: Multimedia Appendix 4 [file jmir_v23i4e22432_app4.pdf]

Multimedia Appendix 4 Perceived usefulness of wanted feature

**Theoretically-based PRISM support[5]**	**Features**	**Patients**												**Patient**	**Clinicians**
		**1**	**2**	**3**	**4**	**5**	**6**	**7**	**8**	**9**	**10**	**11**	**12**		
Information about asthma/ information about available resources	Information about asthma management [Safety net feature]	L			L	U	U	L	U	O	U	O	A	Some patients search for asthma information on the Asthma UK or NHS websites and Google online. Newly diagnosed patients were interested in a broad range of asthma information to learn about how to look after their condition. Patients with more than 10 years asthma experience wanted the IoT system to include asthma information as a 'safety net' so that they can read information if/when they needed. One experienced patient suggested it would be useful to receive customised asthma information as opposed to generic information.	Most of the healthcare professionals commented many patients do not have a good understanding of asthma and so they do not manage their condition well, and it is important to involve professionals to explain the information to patients. Reputable information such as inhaler technique videos, treatment information, why and how the medication should be taken are important to patients' self-management.
Provision of action plan	Teaching patient what to do when their conditions is getting worse [Safety net feature]	O		U	L	U	L	U	U	A *	U	A	A	Most patients want to have an action plan in the system. Some wanted to use a digitalised action plan to remind them what to do when they forgot the agreed actions when their conditions were getting worse.	Most clinicians suggested implementing an action plan in the system to remind about medication adjustment and agreed actions if patients' condition was getting worse.
Regular clinical review	Routine review reminder [Used when needed feature]	O	O		U	A *	A	U		O	A	O	U	Most patients want to be reminded by the system for the yearly review, though whether. they decided to attend depended on their past experience with the doctor/asthma nurse as opposed to a reminder.	Most of the professionals wanted to have a review reminder for patient in order to encourage more patients to attend the consultation.
	Tele/online-consultation [Used when needed feature]	L	U		A	L	L	U	A	L	L	L	U	Most of the patients have used remote consultations with NHS24. Some preferred online or teleconsultation consultation for regular reviews to save travel time. One patient worried that a remote consultation could delay treatment if the professional could not prescribe treatment.	Professionals agreed that tele/online consultations could save patients' travel time and potentially engage some who did not attend regular asthma reviews. However, elderly may find difficulties to use tele/online consultation and they cannot check inhaler patients' inhaler technique by phone.

Monitoring condition with feedback	Logging asthma symptoms, peak flow and medication use<br>[Most wanted feature]	A	A	A<br>*	A	A<br>*	A	A<br>*		A<br>*	O	L	O	Most patients wanted the system to automatically log the data that could reflect their asthma condition (e.g. peak flow, symptoms), inhaler technique, indoor/outdoor environmental data, lifestyle data. They wanted to receive intelligent feedback to learn about their asthma. They wanted to transfer these data to hospital/ practice to reduce the hospital stays or avoid unnecessary regular consultations.	Most clinicians preferred to receive automated logged objective data to help assess a patients' condition and give advice. Some clinicians suggested manual logs might give patients a sense of active involvement to their astha management. Visualising logged data helped patients to understand what affected their asthma and triggered them to think about actions to respond to the changing condition.
	Log reminder<br>[Used when needed feature]	O	A		O	U	A		L	L	L	O	O	A reminder could be useful to remind them to make log when they forgot, however, most patients only log their symptoms/peak flow when their asthma was bad as opposed to logging every day. Thus, they preferred to have a log reminder only when their asthma was bad.	Some clinicians thought reminders were important to remind patients to make log. Others thought that if patients wanted to make log, they would do it without reminder.s One GP suggested using reminder to support patient to make a log may encourage them to take more responsibilities and so engage more in self-management.
	Usual dose alert to patient<br>[Auxiliary feature]	A	A		U	U	L		A	A<br>*	U	O	A	Most patients wanted to know what affected their usual use of medication. Some patients who had not admit to the hospital in the last 12 months wanted the system to alert them when they had taken unusual doses of rescue inhaler.	Most clinicians wanted to know the dose of recuse inhaler taken by patient because it is a warning sign that their asthma may be poorly controlled. They wanted the system to flag up real time overdose warnings to patients and make them stop and think of how to respond to their asthma condition.
Practical support with adherence	Medication reminder<br>[Used when needed feature]	A	A		O	L	A	O	O	L		A	A	Some patients thought reminders were useful to remind them about their medication when they were busy and forgetful. Some (typically over 45 years old) thought they didn't need a reminder because they had their system to remember their medication.	This feature fis or patients but clinicians wanted to know if patients were adherent to preventer medication
	Medication low reminder (blue/brown) and automatically order repeat prescription<br>[Auxiliary feature]	A	O		L	O	A	A<br>*	A<br>*	A<br>*	L	A	A	Most patients wanted to be reminded about the numbers of doses left in the inhaler, especially for the rescue inhaler which they did not take regularly. Although a few devices show the numbers of dose left, they did not automatically prompt re-ordering of medication – a feature patients most wanted on the system.	Most of the clinicians wanted to have a system to flag up a low medication alarm (both preventor and rescue) for patients. Some wanted the system to automatically order the medication for patient, others suggested to ask the patient if they needed to order. Clinicians wanted to an automatic alarm for overordering (as opposed to having to peruse a list of prescriptions at an annual review or practice audit.

	Flu vaccine reminder [Most wanted feature]	O	O	L	A	A *	U	U	L	L	A	U	U	Most patients wanted to have ‘flu vaccine appointment reminder once a year and be alerted when the vaccine was back to stock.	Most clinicians wanted asthma patients to have the ‘flu vaccine injection. Reminders should pop up in both patients and clinicians' system. Patients needed to be reminded to book an appointment complementing alerts on the clinician system highlighting patients who hadn't been vaccinated.
Provision of equipment	---	Depends on how the device is used for different applications												Most patients wanted to try out smart inhalers, smart peak flow meter and smart watch	Clinicians generally interested in how the whole technology system, as opposed to how individual smart device can support patients.
Provision of easy access to support when needed	Panic button for emergency (to the duty doctor in the practice, to NHS 111 or 999) [Auxiliary feature]	L			U	L	O	L	A	U	L	U	U	Patients who had been admitted to hospital in the past 12 months suggested that this feature would be helpful, enabling them to send a text to emergency services because it could be difficult to speak during exacerbations. Some patients thought they didn't need panic button because they would have neighbour to help or they would call NHS24, NHS111 or 999 directly when needed.	Some clinicians suggested there was no need for a duplicate emergency system, but others suggested it provided fast access to emergency services for patients who had brittle, poorly uncontrolled asthma, with a history of intensive admission. A chest physician observed that they want to know patients' hospital admission record as opposed to the numbers of time that they had hit a panic button.
Communication with healthcare professionals	Follow up conversations with GP/ asthma nurse via emails, text messages and Whatsapp [Safety net feature]	A	L	A *	O	L	A	O	A	O	L	O	O	Most patients wanted flexibility to book appointment or raise quick follow up questions. Some patients liked to ‘stay connected' so that could text/ email clinicians when needed. Patients with hearing problems/ was struggled to speak during exacerbation/ and want to communicate with asthma nurse via Whatsapp (e.g. the Asthma UK nurse Whatsapp).	Primary care professional suggested there is a need to shift from annual consultations to a more flexible approach to reviews. Most of the professionals agreed about using text service for quick follow up questions, booking appointments and reminding about reviews. Some suggested that reception could answer administration questions, arrange booking and direct clinical questions to the relevant professional. Resources incurred in non-attendance at face-to-face consultations could be shifted to offer this service.
Training for everyday activities	Air pollution/pollen high alert [Auxiliary feature]	A		O	A	A	A	O	O		A	A	U	Most patients wanted these features on the system. Some used a weather alert app (e.g. BBC weather app) and some were interested to know how the environment affected their asthma.	Some clinicians suggested this is an area in which professionals haven't done enough for asthma patients at the moment. Some wanted environmental information to be an real time education element that could help patients to understand what affected their asthma and think avoiding actions, though one GP thought that, patients may not make changes unless they were severely affected.
	Cross referencing environmental factors and asthma logs to suggest route	A	L	O	O	L	A	O	O	A	O	L	L	Some young patients (16-25 years old) wanted this feature to help them to plan the day.	

	[Auxiliary feature]														Clinicians were less interested in receiving these data, as it could be difficult to interpret alongside other factors that trigger asthma.
	Cross referencing environmental factors and asthma logs to suggest triggers [Auxiliary feature]	A	U		U	U	A	O	U	A *	O	A	O	Some young patients (16-25 years old) wanted this feature to help them to learn about the possible triggers.	
Training for self-management activities	Incorrect inhaler technique alert [Auxiliary feature]	A			O	U	U	L	U	U	A	A	O	Most patients thought it was good to be prompted when their inhaler technique was incorrect. A patient (46-65; >10 years with asthma) felt this feature was not applicable for her but it may be good for her when she was getting older and her hands don't work well.	Most clinicians wanted the system to check patients' inhaler technique automatically and alert patients in real time. Records of the numbers of alerts could be transferred to them for discussion at the next review. Choosing an appropriate device might prevent the need to step up medication when asthma was not controlled
Training for psychological strategies	Breathing exercise [Auxiliary feature]	A		O	A	O	U	O	O	U	L	O	A	Some patients wanted breathing exercise to keep themselves calm and considered it would help their asthma.	Most clinicians were keen to encourage patients to do breathing exercises to improve asthma symptoms.
Social support	---	Most of the patients and clinicians were less interested in this support in the previous study.												One young patient wanted the system to send a message to a friend when she has admitted to the hospital, so she did not need to explain what had happened.	A chest physician suggested a social media page, managed by patients, to enable sharing of asthma experiences and engage patients to look after their asthma.
Lifestyle advice and support	Cross referencing physical activity and asthma logs to suggest fitness trainings [Auxiliary feature]	O			O	O	A	O	O	U	U	L	O	Some patients who exercised frequently wanted the system to learn how exercise impacted on their asthma and to suggest an appropriate fitness regime for their asthma condition. Some patients who used a smart watch wanted the system to be able to connect to their current trackers.	Most clinicians were less interested in looking at patients' exercise intensity, though one GP pointed out that younger patients were interested in sport, and it was good for them to learn how exercise affected their asthma. A chest physician wanted to plan an individualised fitness programme for people with more severe asthma.
	Weight and asthma watching [Auxiliary feature]	O		A	L	O	L		A		L	L	L	Some patients thought being overweight was related to their asthma, and that they would always use this feature. Other thought weight was not a problems for them so they would not be likely to use this feature.	Although most clinicians were less interested in looking at patients' weight, a pharmacist and chest physician wanted to cross reference weight and asthma data to inform an individualised weight management plan.
Note: *top three wanted feature; A: always use; O: often use; U: use when needed; L: less likely to use; grey box: the feature is not applicable to the patients.															
